# Response to dual anti-impulse and lipid-lowering therapy is associated with clinical outcomes in chronic type B aortic syndrome

**DOI:** 10.1007/s11739-026-04348-4

**Published:** 2026-04-20

**Authors:** Paolo Bima, Patrizia Ferrera, Elvira Fanelli, Francesco Revello, Michele La Torre, Emanuele Pivetta, Mauro Rinaldi, Fabio Verzini, Enrico Lupia, Fulvio Morello

**Affiliations:** 1 Dipartimento di Scienze Mediche, Università Degli Studi di Torino, Corso Achille Mario Dogliotti 14, 10126 Turin, Italy; 2 S.C. Medicina d’Urgenza U, Ospedale Molinette, A.O.U. Città della Salute e della Scienza di Torino, Turin, Italy; 3 S.C. Medicina d’Emergenza e d’Urgenza 2, Ospedale S. Giovanni Bosco, Torino, Italy; 4 Scuola di Medicina, Università degli Studi di Torino, Torino, Italy; 5 S.C. Cardiochirurgia U, Ospedale Molinette, A.O.U. Città Della Salute e della Scienza di Torino, Torino, Italy; 6 Dipartimento di Scienze Chirurgiche, Università degli Studi di Torino, Torino, Italy; 7 S.C. Chirurgia Vascolare U, Ospedale Molinette, A.O.U. Città della Salute e della Scienza di Torino, Torino, Italy

**Keywords:** Aorta, Dissection, Chronic, Medical, Treatment, Outcome, Hypertension, Cholesterol

## Abstract

Chronic type B aortic syndrome (cTBAS) is a rare condition characterized by persistent dissection, intramural hematoma or penetrating ulcer within the thoracic descending or thoraco-abdominal aorta. In cTBAS, pathological aortic remodeling leads to a high burden of morbidity and mortality. Guidelines recommend medical treatment with anti-impulse (AI) and lipid-lowering (LL) drugs to improve outcomes, but underlying evidence is sparse, trials are lacking, and feasibility is unknown. We performed a retrospective observational study on consecutive patients with cTBAS followed in a medical clinic from 2021 to 2025, while receiving dual AI/LL therapy with guideline-compliant targets. Response to medical therapy and occurrence of major aortic events (MAEs) were recorded. Thirty-five patients with cTBAS (23 with aortic dissection, 10 with intramural hematoma, and 2 with penetrating aortic ulcer) were followed for a median of 28 months. A MAE occurred in 11 (31%) patients. Demographic and clinical characteristics including systolic/diastolic blood pressure (SBP/DBP), heart rate and lipid levels at baseline were similar in patients who did or did not develop MAEs. During follow-up, patients without MAEs had significant reductions in SBP (*P* = 0.005), total and LDL cholesterol (*P* = 0.002, *P* < 0.001), and increased achievement of SBP < 130 mmHg (*P* = 0.046), and LDL cholesterol < 70 or < 55 mg/dL (*P* < 0.001, *P* = 0.003). All variables were statistically unchanged in patients who developed MAEs. On-target SBP at 6 months and LDL cholesterol at 12 months were associated with protection from MAEs (hazard ratio 0.19, *P* = 0.014, and 0.15, *P* = 0.004, respectively). In conclusion, results provide proof-of-concept that in cTBAS, strict dual AI/LL medical therapy is feasible and associated with favorable outcomes.

**Supplementary Information:**

The online version contains supplementary material available at 10.1007/s11739-026-04348-4.

## Introduction

Acute aortic syndrome (AAS), including aortic dissection (AD), intramural aortic hematoma (IMH) and penetrating aortic ulcer (PAU), is a life-threatening disease category affecting 3 to 6 per 100,000 person-years [1–3]. Acute phase treatment of AAS differs based on anatomic involvement and clinical picture. Stanford type A AAS, characterized by ascending aorta involvement, is treated with emergency surgical intervention. Stanford type B AAS, which involves the thoracic descending or thoraco-abdominal aorta while sparing the ascending aorta, is treated with intensive medical therapy aiming at tight blood pressure (BP), heart rate (HR) and pain control, while emergency surgical or endovascular intervention is due only in complicated cases [4, 5].

In patients surviving > 14 days from AAS onset, persistence of AD, IMH or PAU within the descending thoracic or thoraco-abdominal aorta leads to development of chronic Stanford type B aortic syndrome (cTBAS). This condition is characterized by incessant pathological aortic remodeling due to inflammation, extracellular matrix degradation, loss of vascular smooth muscle cells and thrombosis [6, 7]. Such processes pave the way for aneurysm formation, AAS recurrence, aortic rupture and therefore, subsequent interventions. Within 5 years, 63%–73% of patients with type B AD develop an aortic aneurysm. At 10 years, intervention rate is 20–50%, and mortality rate is 30–50%, with differences according to subtype [8–10]. Preventing this burden of morbidity and mortality is a clinically unmet need, with medical therapy, patient surveillance and patient selection for early thoracic endovascular repair (TEVAR) representing key albeit controversial elements [11, 12].

The mainstay therapeutic recommendation for cTBAS is represented by long-term anti-impulse pharmacological restraint of the rate of aortic pressure rise over time (dP/dT), representing the main cause of aortic wall shear stress [13]. This is obtained through coupling of both BP and HR reduction. For cTBAS, guidelines indicate targets of systolic BP (SBP) < 130 mmHg and HR of 60–70 beats per minute (bpm) [4, 5]. In clinical practice, this is usually obtained by an association therapy including a beta-blocker drug with other hypotensive drugs, as needed. In animal models of aortic dissection, blockade of beta-adrenergic or angiotensin II signaling has shown beneficial effects [14]. However, supporting evidence in humans from clinical studies is sparse and conflicting [15]. Randomized controlled trials are not available, and only few observational studies have reported that long-term therapy with beta-blockers, ACE inhibitors/ARBs or calcium channel blockers is associated with favorable outcomes in cTBAS [16–19]. In a recent survey across 7 countries, substantial variation in therapeutic targets, reporting standards, and management has been highlighted [20].

Another component of long-term medical treatment of cTBAS is represented by lipid-lowering (LL) therapy. In patients with underlying atherosclerotic aortic disease, LL drugs may promote favorable remodeling of atherosclerotic plaques. In addition, these drugs (especially statins) may provide pleiotropic benefits such as reduction of tissue inflammation, restraint of fibrotic remodeling and protection from vascular smooth muscle cell loss, even in non-atherosclerotic aortas [21, 22]. However, the benefits of LL therapy in human cTBAS have been shown only in few observational studies [23–26]. Furthermore, lipid-lowering targets tailored to cTBAS are not specified by guidelines. Assuming high or very-high cardiovascular risk for cTBAS, recommended LDL cholesterol targets are < 70 mg/dL (ACC/AHA and JAS guidelines) or < 55 mg/dL (ESC/EAS guidelines) [27–29].

Herein, we performed an observational proof-of-concept retrospective study, evaluating outpatients with cTBAS managed by a specialized clinic. All patients were treated with a best medical therapy bundle including anti-impulse and LL drugs, aimed at guideline-compliant targets for BP, HR and lipid levels. The working hypothesis was that favorable response to medical therapy is associated with better clinical outcomes.

## Methods

### Study design and setting

This was a single-center retrospective observational study conducted at the Molinette Hospital, A.O.U. Città della Salute e della Scienza di Torino, an interregional (Piemonte, Valle d’Aosta) referral hub for complex aortic diseases. The study protocol conformed to the Declaration of Helsinki and was approved by the local institutional Ethics Committee (42/2020, prot. 0029448). All participants provided informed consent, and data were collected anonymously, in compliance with data protection regulations.

### Study patients

The study included consecutive outpatients affected by cTBAS, referred for best medical therapy to a specialized clinic of the Molinette Hospital (Torino, Italy) between March 2021 and May 2025. Referrals were decided based on clinical reasoning by physicians who were not involved in the study. Referred patients were considered eligible if they had a confirmed diagnosis of cTBAS, which included persisting dissection, intramural hematoma or penetrating ulcer within the descending thoracic or thoraco-abdominal aorta. Both primary and residual forms after a surgical repair of Stanford type A AAS were eligible. Chronicity of cTBAS was defined by more than 30 days from AAS onset.

The diagnosis of cTBAS was confirmed by a senior physician expert in cardiovascular medicine and by a vascular surgeon expert in aortic diseases, who had access to full medical data and imaging. Exclusion criteria were age < 18 years, traumatic etiology, AAS onset ≤ 30 days, patient’s refusal to participate, unwillingness or impossibility to show up at follow-up visits and incomplete clinical data. All consecutive patients satisfying inclusion criteria and without exclusion criteria were enrolled in the study independent of previous medical prescriptions, current pharmacological treatment and response to therapy at day 0 in terms of BP, HR and lipid levels.

### Clinical management

The clinic implemented a pre-defined structured outpatient surveillance program which included scheduled medical visits and serial multimodal vascular imaging, compliant with available guidelines. The clinical protocol involved a first visit, which represented day 0 of patient’s follow-up for the present analyses, subsequent visits scheduled after 6 and 12 months, and annually thereafter unless clinically needed. For patients enrolled after hospital admittance for AAS, the first outpatient visit was generally scheduled within 3 months from hospital discharge, based on feasibility and clinical reasoning. When in-person visits were not feasible, follow-up was conducted via telephone contact.

Overall clinical management of enrolled patients, including indications for surgical or endovascular interventions, was independent from patient enrollment in the present study. For medical therapy, however, the clinical policy of the surveillance program was to provide all patients with prescription of best medical therapy according to guidelines including anti-impulse therapy with target office BP values < 130/80 mmHg and HR < 60 beats per minute (bpm), and LL therapy aiming at LDL cholesterol < 55 mg/dL.

### Data collection and variables

Clinical and outcome data were collected through manual review of all available hospital and clinic records by research personnel not involved in data analysis. Demographic, clinical and imaging data were collected anonymously in a structured dataset compiled using REDCap®. Demographic data included age, gender and body mass index. Clinical data included type of cTBAS diagnosis and previous AAS, “classic” cardiovascular risk factors (including hypertension, diabetes mellitus, dyslipidemia, smoking, drug use), risk factors for AAS (connective tissue disease, family history), history of aortic disease (aortic valve disease, previous aortic surgery or endovascular procedure, aortic aneurysm or aortic grafts), cardiovascular comorbidities (including coronary artery disease, peripheral artery disease, cerebrovascular disease), and other relevant comorbidities (chronic kidney disease, connective tissue disorder). Additional data included type and dose of pharmacological therapy.

The following clinical data were registered based on report by the treating physician at each time point: office SBP, diastolic BP (DBP) and HR values, and home self-reported BP control. Office BP was measured with a manual sphygmomanometer, following guideline indications. Per guidelines, office and self-reported home BP control was defined as optimal if < 130/80 mmHg. For HR, optimal control was defined if < 60 beats per minute (bpm). The patient’s lipid profile registered in the study included total cholesterol, LDL cholesterol, HDL cholesterol and triglycerides. Additional data included selected blood tests obtained within 3 months of patient enrollment (creatinine, C-reactive protein and D-dimer). All missing values were left as non-imputed.

### Outcomes

The primary outcome was a composite of major aortic events (MAEs), defined as development of any of the following: death from any aortic cause, aortic rupture, new AAS, aortic dilatation defined as transverse diameter increase > 5 mm/year or maximal aortic diameter > 55 mm at any aortic level, new aortic intervention (either surgical or endovascular, at any level) or new aorta-related malperfusion or ischemic organ damage. In case of death from unknown cause after review of all available data, the case was considered as a MAE, applying worst-case-scenario. In case of repeated MAEs during the follow-up, the earliest was considered for all time-dependent analyses. The secondary outcome was a composite of major non-aortic cardiovascular events, defined as any of the following: death from cardiovascular non-aortic causes, acute coronary syndrome, cerebrovascular event (stroke or transient ischemic attack), new carotid revascularization, new coronary revascularization, limb ischemia or peripheral arterial revascularization. Overall mortality, including that resulting from non-aortic or cardiovascular conditions, was also annotated. Outcome adjudication was performed by two expert physicians who were not involved in data analysis and who had full access to the clinical data. In case of discordance, final adjudication was done after case discussion.

### Statistical analysis

Continuous variables were reported as median and interquartile range (IQR, presented as 25th to 75th percentile value). Categorical variables were reported as counts and percentages. Statistical comparisons between groups were made using Wilcoxon rank-sum exact test for continuous variables, and Pearson’s chi-square or Fisher’s exact test for categorical values. Comparison of repeated measures in the same group was made using non-parametric Friedman's Two-Way Analysis of Variance by Ranks and Wilcoxon rank-sum exact test with Holm-Bonferroni correction for continuous variables, and Cochran’s Q test and McNemar test with Holm-Bonferroni correction for categorical values.

Cox proportional hazards regression analysis (both univariate and bivariate adjusting for the presence of abdominal aortic aneurysm) was used to assess the association between selected variables and MAEs. Hazard ratios with corresponding 95% confidence intervals (CIs) were estimated. Kaplan–Meier survival curves were reported, evaluating the association between selected variables and MAEs.

Since this was an observational study performed on consecutive eligible patients with a rare condition and without an external control group, sample size calculations were not performed. Statistical analysis was performed using MedCalc® Statistical Software version 23.3.4 (MedCalc Software Ltd, Ostend, Belgium; https://www.medcalc.org; 2025), SPSS Statistics for Windows, ver. 27.0, (IBM Corp., Chicago, IL, USA), and R Software v4.3.1 for Mac OS (R Foundation for Statistical Computing, Vienna, Austria). A two-sided *P*-value < 0.05 was considered statistically significant.

## Results

### Patient characteristics

**Fig. 1:**
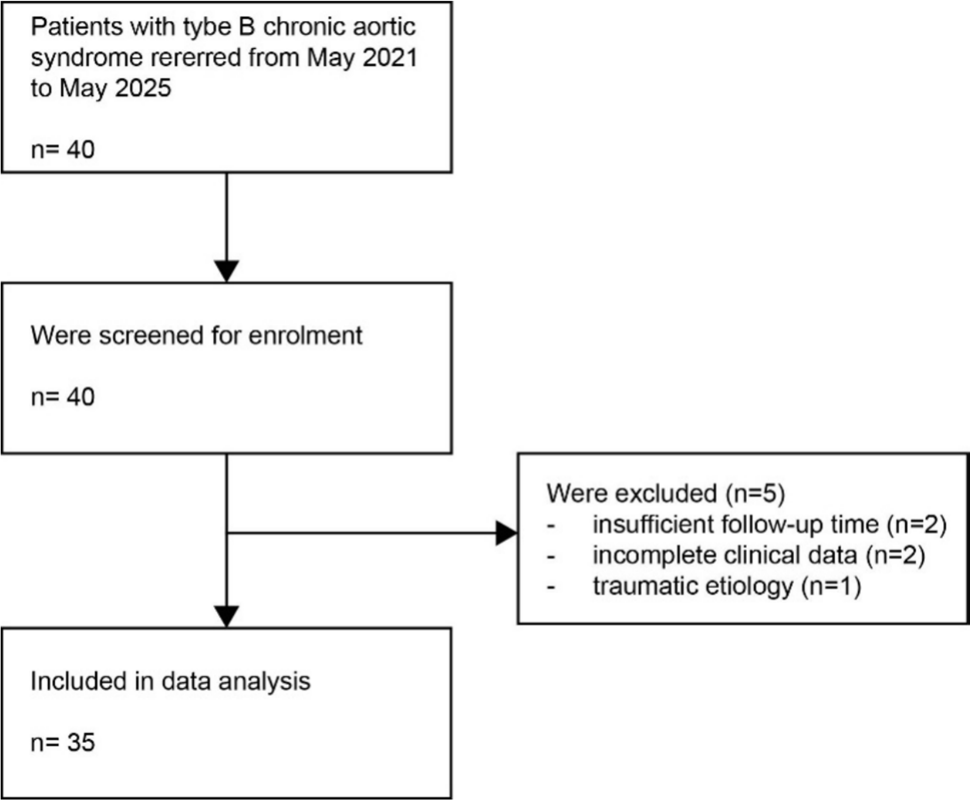
Flow diagram of the study

Forty patients with cTBAS were screened for eligibility and 35 were further analyzed (Fig. 1). Nineteen (54.3%) developed primary AAS from January 2021 to January 2025, and the other 16 patients had earlier primary AAS. In the study period, 165 type A AASs and 41 type B AASs were admitted to the study hospital through the Emergency Department; 132 and 38 of them, respectively, were discharged alive. cTBAS subtypes included 23 (65.7%) ADs, 10 (28.6%) IMHs, and 2 (5.7%) of PAUs. The AAS leading to cTBAS was a primary type B AAS in 28 (80%) patients, and a previous surgically treated type A AAS in 7 (20%) patients (6 AD and 1 IMH). The median time from AAS diagnosis to study enrollment was 536 days. Sixty-three percent of patients were male, and the median age was 60 (53.5–66) years. Demographic and clinical characteristics of study patients are summarized in Table 1.

**Table 1:** Demographic and clinical characteristics of study patients at baseline

Variables	All patients *n* = 35	Without MAE *n* = 24	With MAEs *n* = 11	*P*-value
Patient characteristics				
Age, years	60 (54–66)	59.5 (54–63)	63 (53–75)	0.534
Male sex	22 (62.9%)	15 (62.5%)	7 (63.6%)	0.948
BMI, kg/m^2^	26.6 (24.2–31.5)	26.1 (23.6–30.7)	28.1 (25.8–32.3)	0.517
Hypertension	35 (100.0%)	24 (100.0%)	11 (100.0%)	1
Dyslipidemia	35 (100.0%)	24 (100.0%)	11 (100.0%)	1
Diabetes mellitus	1 (2.9%)	1 (4.2%)	0 (0.0%)	0.512
Active smoking	10 (28.6%)	6 (25.0%)	4 (36.4%)	0.489
Coronary artery disease	5 (14.7%)	3 (12.5%)	2 (20.0%)	0.574
Cerebrovascular disease	4 (11.4%)	3 (12.5%)	1 (9.1%)	0.768
Peripheral artery disease	4 (11.4%)	2 (8.3%)	2 (18.2%)	0.395
Chronic kidney disease	9 (25.7%)	5 (20.8%)	4 (36.4%)	0.329
Connective tissue disease	1 (2.9%)	1 (4.2%)	0 (0.0%)	0.492
Family history of acute aortic syndrome	2 (5.7%)	2 (8.3%)	0 (0.0%)	0.324
Aortic valve disease	4 (11.8%)	4 (16.7%)	0 (0.0%)	0.169
Thoracic aortic aneurysm	13 (37.1%)	7 (29.2%)	6 (54.5%)	0.149
Abdominal aortic aneurysm	3 (8.6%)	0 (0.0%)	3 (27.3%)	0.007
Time from acute aortic syndrome*, days	536 (66–1731)	550 (104–1459)	270 (38–2181)	0.612
Previous aortic intervention				
Thoracic aortic surgery	10 (29.4%)	7 (30.4%)	3 (27.3%)	0.850
Abdominal aortic surgery	1 (3.0%)	0 (0.0%)	1 (9.1%)	0.151
Thoracic endovascular aortic repair	8 (22.9%)	6 (25.0%)	2 (18.2%)	0.656
Abdominal endovascular aortic repair	0 (0.0%)	0 (0.0%)	0 (0.0%)	1
Vital parameters at baseline				
Systolic blood pressure, mmHg	130 (120–140)	130 (120–133)	130 (123–140)	0.588
Diastolic blood pressure, mmHg	80 (70–85)	80 (70–85)	80 (75–85)	0.420
Heart rate, bpm	60 (60–70)	60 (58–65)	70 (60–72)	0.117
Blood tests				
Creatinine (mg/dL)	1.04 (0.80–1.19)	1.03 (0.80–1.15)	1.07 (0.82–1.34)	0.594
C-reactive protein (mg/L)	4 (2.0–10.4)	4.4 (2.4–12.2)	3.5 (1.6–6.0)	0.662
D-dimer (ng/mL)	3410 (1766–5715)	3641 (2277–5632)	2519 (1766–6015)	0.799
Total cholesterol (mg/dL)	154 (132–181)	148.5 (132–205)	157 (132–165)	0.320
LDL cholesterol (mg/dL)	83 (69–119)	85 (68–130)	82 (73–92)	0.434
HDL cholesterol (mg/dL)	51 (41–55)	54 (44–58)	45 (38–52)	0.094
Triglycerides (mg/dL)	95 (80–127)	96 (77–135)	95 (81–111)	0.499

*MAE(s)* Major aortic event(s). Age, BMI and time from onset of acute aortic syndrome, are expressed as median (25th–75th percentile). The other variables are presented as n (%)

^*^Time from onset of the first acute aortic syndrome to enrollment in the present study, i.e. to follow-up

### Response to medical therapy

The median follow-up time was 839 (640–1256) days. All patients were treated with anti-impulse and lipid-lowering drugs, as detailed in Table 2. Antihypertensive and LL regimens were intensified over time (*P* = 0.032 and *P* < 0.001, respectively). BP, HR, and lipid levels measured during follow-up are shown in Table 3. LDL cholesterol decreased over time, from a median value of 83 mg/dL at baseline to 56 mg/dL after 12 months (*P* = 0.003), corresponding to a median −32.5% reduction. Also triglycerides decreased from 95 mg/dL to 85 mg/dL, corresponding to a median −10.5% reduction (*P* = 0.025).

**Table 2:** Antihypertensive and lipid-lowering therapy in study patients

Variable	Baseline *n* = 35	6 months *n* = 33^¶^	12 months *n* = 34^§^	*P*-value
Antihypertensive drugs				
Number of drug classes/patient	3 (2–4)	3 (3–4)	3 (3–4)	0.032
Number of anti-impulse drugs/patient	2 (2–3)	3 (2–3)	3 (2–3)	0.171
Beta-blocker	32 (91.4%)	31 (93.9%)	29 (87.9%)	0.370
Calcium channel blocker	24 (68.6%)	25 (75.8%)	27 (81.8%)	0.115
RAS inhibitor (ACEi, ARBs)	27 (77.1%)	27 (81.8%)	27 (81.8%)	0.717
Diuretic	12 (34.3%)	13 (39.4%)	16 (48.5%)	0.417
Aldosterone receptor antagonist	9 (25.7%)	9 (27.3%)	11 (33.3%)	0.867
Alpha-blocker	8 (22.9%)	6 (18.2%)	6 (18.2%)	1
Other drugs	2 (5.7%)	1 (3%)	2 (6.1%)	0.370
Lipid lowering drugs				
Number of drug classes/patient	1 (0–1)	2 (1–2)	2 (1–2)	< 0.001*†‡
Statin	21 (60%)	28 (84.8%)	29 (87.9%)	0.003*†
Ezetimibe	5 (14.3%)	17 (51.5%)	20 (60.6%)	< 0.001*†
Bempedoic acid	0 (0%)	4 (12.1%)	6 (18.2%)	0.009*†
PCSK9 inhibitor	1 (2.9%)	1 (3%)	1 (3%)	0.368

The number of drug classes and of anti-impulse drugs per patient are presented as median and interquartile range (in parenthesis). The other variables are presented as n (%). ^**¶**^Data missing for 2 patients with data available at 12 months. ^**§**^Data missing for 1 dead patient. Anti-impulse drugs include β-blockers, calcium channel blockers and renin-angiotensin system (RAS) inhibitors. *P*-value refers to Friedman rank sum test or Cochran’s Q test. *P < 0.05 for paired comparison between baseline and 6 months. ^†^*P* < 0.05 for paired comparison between baseline and 12 months. ^‡^*P* < 0.05 for paired comparison between 6 and 12 months

**Table 3:** Blood pressure and lipid levels in study patients

Variable	Baseline	6 months	12 months	*P*-value
Systolic blood pressure, mmHg	130 (120–140) *N* = 35	120 (115–130) *N* = 31	125 (113–130) *N* = 30	0.062
Diastolic blood pressure, mmHg	80 (70–85) *N* = 35	70 (70–80) *N* = 31	80 (70–80) *N* = 30	0.069
Heart rate, bpm	60 (60–70) *N* = 35	60 (55–68) *N* = 31	60 (55–65) *N* = 30	0.863
Total cholesterol, mg/dL	154 (132–181) *N* = 35	134 (118–158) *N* = 27	126 (110–136) *N* = 33	0.010*†
LDL cholesterol, mg/dL	83 (69–119) *N* = 35	61 (52–78) *N* = 26	56 (46–66) *N* = 33	0.003*†
HDL cholesterol, mg/dL	51 (41–55) *N* = 35	50 (47–58) *N* = 26	53 (44–59) *N* = 33	0.388
Triglycerides, mg/dL	95 (80–127) *N* = 35	96 (86–119) *N* = 27	85 (70–110) *N* = 33	0.025

Values are presented as median and interquartile range (in parenthesis). N indicates the number of patients with available data at each time point. *P*-value refers to Friedman rank sum test. *P < 0.05 for paired comparison between baseline and 6 months; ^†^*P* < 0.05 for paired comparison between baseline and 12 months

The proportions of patients at guideline targets during the study are detailed in Supplementary Table 1 (SBP, DBP, HR) and in Supplementary Table 2 (lipid levels). Individuals at target for SBP were 40% at baseline and 53.3% after 12 months (*P* = 0.144), for DBP 37.1% and 46.7% (*P* = 0.050), and for HR 25.7% and 36.7% (*P* = 0.695). Individuals at target for LDL cholesterol *per* ACC/AHA (< 70 mg/dL) or ESC/EAS guidelines (< 55 mg/dL) increased from 28.6% and 8.6% at baseline, to 75.8% (*P* < 0.001) and 42.4% (*P* = 0.002), respectively, after 12 months.

### Outcomes

During follow-up, 11 (31.4%) patients had at least one MAE. Total MAEs, including multiple MAEs, were 3 (8.6%) deaths from aortic disease, 1 (2.9%) new AAS, 2 (5.7%) malperfusion syndromes, 5 (14.3%) aortic dilatations, and 5 (14.3%) aortic interventions. 1 patient (2.9%) had a non-aortic cardiovascular event, represented by new non-aorta-related peripheral artery disease.

Baseline demographic and clinical characteristics of patients with and without MAEs are shown in Table 1. All baseline variables, including SBP, DBP, HR and lipid levels, did not statistically differ between patients who subsequently developed or did not develop MAEs, except for abdominal aortic aneurysm at inclusion. This condition was more prevalent in patients who developed MAEs and was statistically associated with development of MAEs with a hazard ratio of 12.5 (95% CI, 2.7–58.2; *P* = 0.001).

### Association with medical response

**Fig. 2:**
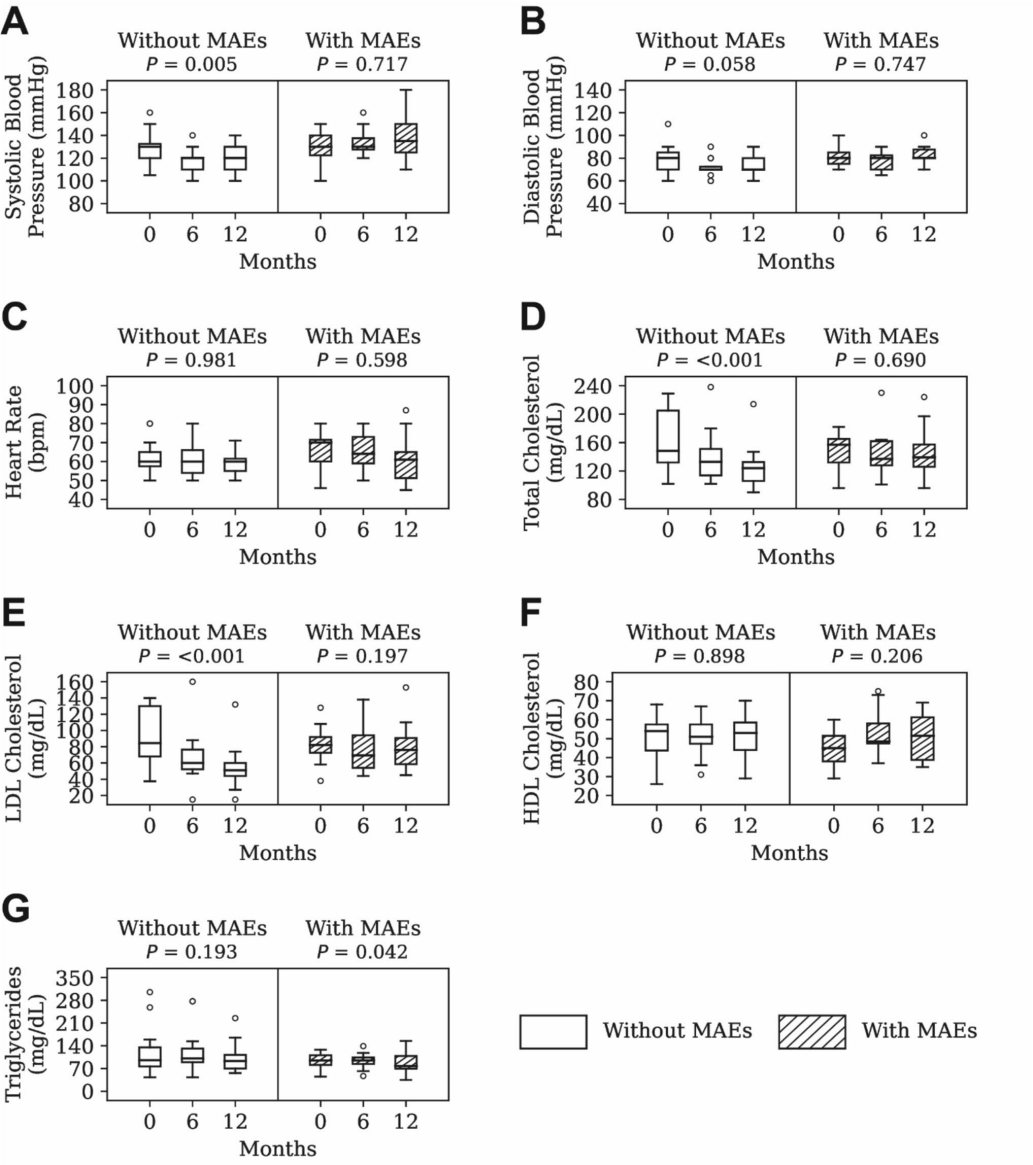
Box-and-whisker plots representing levels of blood pressure (**A,B**), heart rate (**C**) and lipid levels (**D**, **E**, **F**, **G**) at baseline (0 months) and during follow-up (6 and 12 months), in study patients without and with major aortic events (MAEs). Boxes represent the 25th quartile, median, 75th quartile, and outlier values are presented. P-values in each patient group were obtained with Friedman two-way analysis of variance by ranks

The distributions of SBP, DBP, HR and lipid levels during clinical follow-up, in patients with and without MAEs, are represented in Fig. 2. In patients who developed MAEs, SBP, DBP, HR and lipid levels were statistically unchanged over time. In this patient group, the proportions of individuals at target for SBP, DBP and HR were statistically unchanged during follow-up (Supplementary Table 1), whereas an increasing trend was observed in the proportion of patients reaching the LDL cholesterol target *per* ACC/AHA definition (18.2% to 40%, *P* = 0.05; Supplementary Table 2). Conversely, in patients without MAEs, significant changes were observed in SBP (*P* = 0.005), total cholesterol (*P* < 0.001) and LDL cholesterol (*P* < 0.001). Accordingly, the proportions of patients at target for SBP (41.7% to 60%, *P* = 0.046), DBP (41.7% to 65%, *P* = 0.050) and LDL cholesterol < 70 mg/dL (33.3% to 91.3%, *P* < 0.001) or < 55 mg/dL (8.3% to 56.5%, *P* = 0.003), increased during the follow-up in patients without MAEs. The number of anti-impulse and LL drug classes at baseline, 6 and 12 months did not differ between patients with and without MAEs (Supplementary Table 3), while the number of LL drug classes increased significantly over time, in both groups.

After 6 months, in patients with and without MAEs, SBP was at target in 27.3% *vs* 80% individuals (*P* = 0.004), and DBP was at target in 36.4% *vs* 75% (*P* = 0.035), respectively. After 12 months, LDL cholesterol was at target < 70 mg/dL in 40% *vs* 91.3% (*P* = 0.002), and < 55 mg/dL in 10% *vs* 56.5% (*P* = 0.013), respectively.

**Fig. 3:**
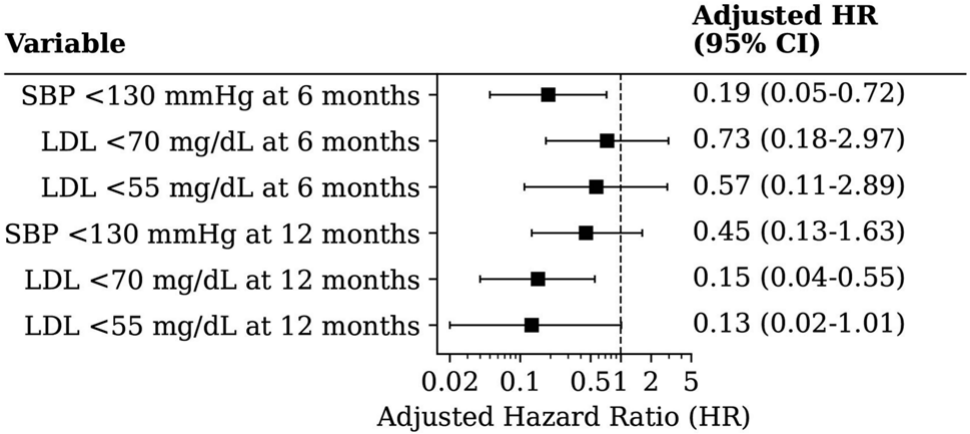
Forest plot summarizing the adjusted hazard ratio for major aortic events, associated with the indicated medical response variables. The HR is presented in log scale. Values in brackets represent the lower and upper limits of the 95% confidence interval

**Fig. 4:**
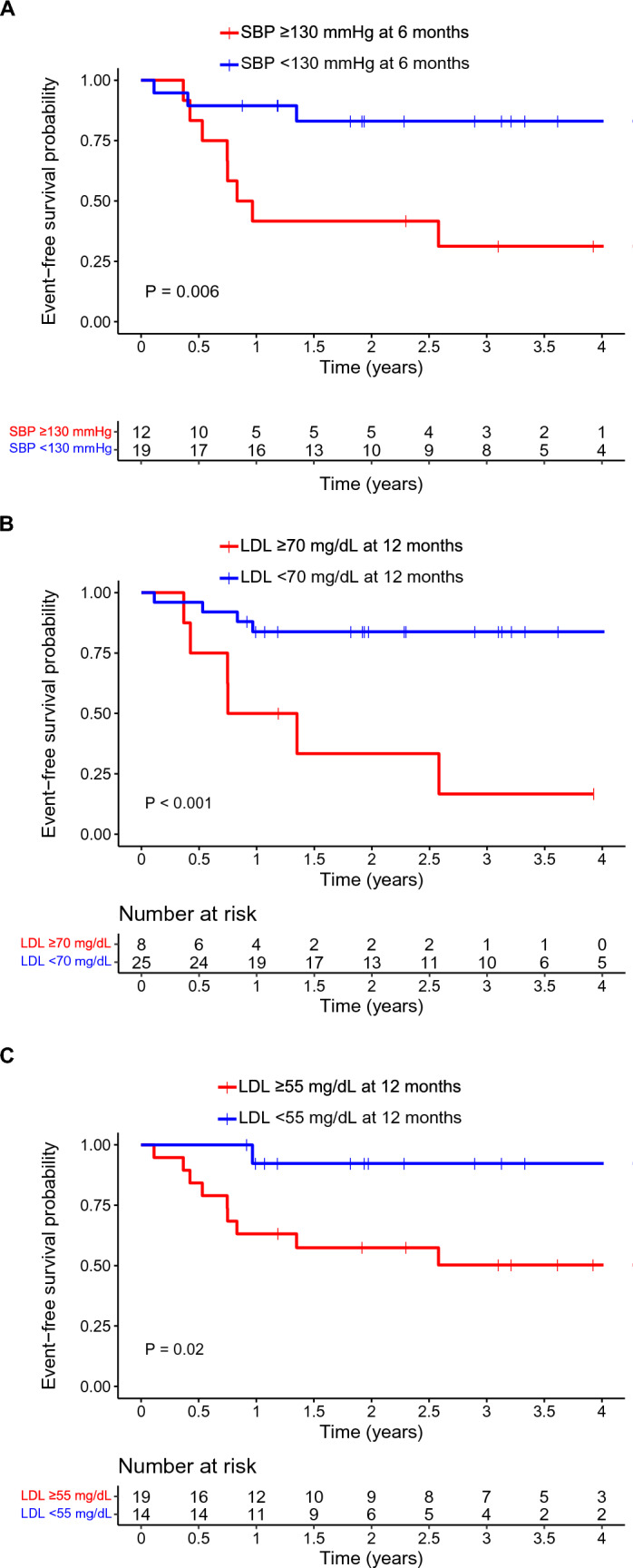
Major aortic event-free survival curve of study patients classified according to achievement of pharmacological goals. **A** Therapeutic target: office systolic blood pressure (SBP) < 130 mmHg at 6 months. **B** Therapeutic target: LDL cholesterol < 70 mg/dL at 12 months. **C** Therapeutic target: LDL cholesterol < 55 mg/dL at 12 months. P-values were obtained with the Log-rank test

The results of Cox-regression analysis are summarized in Fig. 3. SBP < 130 mmHg at 6 months (hazard ratio 0.19, *P* = 0.014) and LDL cholesterol < 70 mg/dL at 12 months (hazard ratio 0.15, *P* = 0.004) were both associated with protection from MAEs. The association remained significant after adjustment for the presence of an abdominal aortic aneurysm (Supplementary Fig. 1). Event-free survival curves for patients classified according to achievement of SBP or LDL targets are shown in Fig. 4.

## Discussion

Current expert and guideline recommendations for long-term anti-impulse and LL therapies in cTBAS are poorly evidence-based, as observational studies have provided conflicting results. Furthermore, clinical trials are lacking, beneficial effects have mostly been described in animal or basic science studies, and heterogeneous reference, management and implementation standards have been reported [14, 20]. Herein, we provide a hypothesis-generating study suggesting that in cTBAS outpatients under supervision of a specialized medical clinic, pharmacological treatment strictly targeting BP, HR and LDL cholesterol may be associated with protection from MAEs. In patients who developed MAEs, BP, total and LDL cholesterol levels were unchanged during follow-up, indicating that conversely, failure to obtain satisfactory medical control may be associated with increased risk of MAEs. Since all patients received the same prescription and follow-up protocol, differences in outcomes appear related to individual response to medical therapy. Nonetheless, given the observational nature of the study, causality cannot be inferred, nor could adjustment for potential confounders be performed.

Results suggest that in current practice, optimal control of BP, HR and lipid levels in patients with cTBAS still represents a challenge and that improved awareness is warranted. At study enrollment, patients at target for BP, HR and LDL cholesterol were a net minority. In terms of feasibility, the study shows that an intensive medical program, implying a net increase in the number of drug prescriptions, can indeed improve response towards guideline-compliant targets and possibly be associated with favorable outcomes. However, the proportion of patients not at target was still significant after 12 months, especially for anti-impulse targets and for LDL cholesterol < 55 mg/dL.

Previous pilot translational and small observational studies in patients with cTBAS showed that anti-impulse and LL therapy seem to mitigate pathological aortic remodeling [14, 23–26]. In our study, the beneficial effects of this dual therapy seemed to develop within the first months, reaching statistical evidence within 6 to 12 months. Results reinforce the importance of early adoption or development for patients with cTBAS, of intensive programs dedicated to cardiovascular risk factor control and serial outpatient control, in the attempt to improve their clinical outcomes. In this study, however, the benefits of medical therapy were found also in patients with long-lasting cTBAS, as median time from AAS to study enrollment was 18 months. Unfortunately, a subgroup analysis by “chronicity” could not be performed given the low numbers in the groups. Considering the biology of aortic remodeling, we speculate that an earlier start of targeted medical therapy might lead to better outcomes. Nonetheless, whether these strict targets are beneficial for all cTBAS and should be applied regardless of the phenotype and amount of aortic atherosclerosis, remains to be understood in further studies.

Given the study design, causality cannot be unraveled, and results are hypothesis-generating, with two possible and potentially coexisting explanations. In line with translational studies, antihypertensive and LL therapies may protect from negative aortic wall remodeling following a dose–response relationship. Only in patients with a significant drop in BP and in LDL cholesterol levels, protective effects on the aortic wall are elicited, even after adjusting for the presence of an abdominal aortic aneurysm. An alternative explanation is that therapeutic response is underpinned by a combination of therapeutic adherence, additional lifestyle variables (*e.g.* dietary habits, metabolic phenotype) and genetic determinants (of both therapeutic response and aortic disease), which were not evaluated in our study.

Due to small sample size, the present study does not inform on the relative importance of therapeutic bundle components in MAE prevention. It is therefore unknown whether anti-impulse and LL therapy are both needed to boost aortic protection, or whether one of the two may be sufficient, or even superior. For the same reason, comparison between drug classes, or between medical therapy alone and medical therapy combined with aortic interventions, is not possible.

The current study has several limitations. First, this was an observational single-center study with a small sample size, which limits external validity and cannot assess causality. Since patients were selected based on their adequacy to attend an outpatient clinic, the cohort likely overrepresents younger and more motivated patients with reasonably high therapeutic compliance. We cannot exclude additional selection bias, since referrals were made by physicians not involved in the present study. Second, pharmacological compliance was not directly assessed through specific questionnaires or tests. It is possible that patients with reduced clinical response were less adherent to prescriptions. Third, BP and HR levels were only measured as office values, while more informative data from 24 h monitoring, which would account for daily variability and white-coat effect, was not available. Finally, the small sample size does not allow subgroup analyses to evaluate the impact of the different components of the therapeutic bundle and adjustment for confounders in multivariate analyses.

In conclusion, the present study provides proof-of-concept that in cTBAS, long-term combined medical therapy with anti-impulse and LL drugs applying ambitious guideline-compliant targets is feasible, and that medical response may be associated with favorable outcomes. Further multicenter studies are warranted to confirm these pilot results on larger patient cohorts.

## Supplementary Information

Below is the link to the electronic supplementary material.

Supplementary file1 (PDF 155 KB)

## Data Availability

The datasets generated and analysed during the current study are not publicly available due to patient privacy and ethical restrictions. De-identified aggregate data are available from the corresponding author on reasonable request.
