# Supplementary material for: Response to dual anti-impulse and lipid-lowering therapy is associated with clinical outcomes in chronic type B aortic syndrome
Source: Intern Emerg Med. 2026 Apr 20;21(5):1635–46. doi: 10.1007/s11739-026-04348-4 (PMC13452837; doi:10.1007/s11739-026-04348-4)
Supplement: Supplementary file 1 [file 11739_2026_4348_MOESM1_ESM.pdf]

SUPPLEMENTARY MATERIAL

Response to dual anti-impulse and lipid-lowering therapy is associated with clinical outcomes in chronic type B aortic syndrome

Paolo Bima, Patrizia Ferrera, Elvira Fanelli, Francesco Revello, Michele La Torre, Emanuele Pivetta, Mauro Rinaldi, Fabio Verzini, Enrico Lupia, Fulvio Morello

SUMMARY

Supplementary Table 1. Page 2

Supplementary Table 2. Page 3

Supplementary Table 3. Page 4

Supplementary Figure 1. Page 5

Supplementary Table 1. Patients at target for blood pressure and heart rate during the study follow-up.

**Variables**	**Overall**	**Without major aortic events**	**With major aortic event(s)**	***P*-value[a]**
**Systolic blood pressure at target (<130 mmHg)**				
baseline	14 (40.0%)	10 (41.7%)	4 (36.4%)	0.766
6 months	19 (61.3%)	16 (80%)	3 (27.3%)	0.004
12 months	16 (53.3%)	12 (60%)	4 (40%)	0.301
P-value[b]	0.144	0.046	0.472	
**Diastolic blood pressure at target (<80 mmHg)**				
baseline	13 (37.1%)	10 (41.7%)	3 (27.3%)	0.413
6 months	19 (61.3%)	15 (75%)	4 (36.4%)	0.035
12 months	14 (46.7%)	13 (65%)	1 (10%)	0.004
P-value[b]	0.050	0.050	0.368	
**Home blood pressure at target (<130/80 mmHg)**				
baseline	28 (84.8%)	20 (87.0%)	8 (80.0%)	0.609
6 months	30 (100.0%)	19 (100.0%)	11 (100.0%)	1
12 months	28 (90.3%)	21 (95.5%)	7 (77.8%)	0.131
P-value[b]	0.174	0.368	0.264	
**Heart rate at target (<60 bpm)**				
baseline	9 (25.7%)	7 (29.2%)	2 (18.2%)	0.490
6 months	10 (32.3%)	7 (35%)	3 (27.3%)	0.660
12 months	11 (36.7%)	7 (35%)	4 (40%)	0.789
P-value[b]	0.695	0.513	0.449	

Legend. MAEs: Major aortic event(s). Values are reported as absolute number and % of column (in brackets). [a]*P*-value for comparison between patients with and without major aortic events. [b]*P*-value of Cochran's Q test for comparison between time points, in the corresponding patient group. **P* <0.05 for paired comparison between baseline and 6 months; †*P*<0.05 for paired comparison between baseline and 12 months (McNemar test with Holm-Bonferroni correction).

Supplementary Table 2. Patients at LDL cholesterol target during the study follow-up.

Variables	Overall	Without major aortic events	With major aortic event(s)	*P*-value[a]
**LDL cholesterol at ACC/AHA target (<70 mg/dL)**				
baseline	10 (28.6%)	8 (33.3%)	2 (18.2%)	0.357
6 months	15 (57.7%)	11 (61.1%)	4 (50%)	0.597
12 months	25 (75.8%)	21 (91.3%)	4 (40%)	0.002
P-value[b]	<0.001*†	<0.001†	0.050	
**LDL cholesterol at ESC/EAS target (<55 mg/dL)**				
baseline	3 (8.6%)	2 (8.3%)	1 (9.1%)	0.941
6 months	10 (38.5%)	8 (44.4%)	2 (25%)	0.347
12 months	14 (42.4%)	13 (56.5%)	1 (10%)	0.013
P-value[b]	0.002*†	0.003*†	0.135	

Legend. Values are reported as absolute number and % of column (in brackets). [a]*P*-value for comparison between patients with and without major aortic events. [b]*P*-value of Cochran's Q test for comparison between time points, in the corresponding patient group. **P* <0.05 for paired comparison between baseline and 6 months; †*P*<0.05 for paired comparison between baseline and 12 months (Mc Nemar test with Holm-Bonferroni correction).

Supplementary Table 3. Number of anti-impulse drug classes (ACEi/ARB, beta-blockers and calcium antagonists) and of lipid-lowering drug classes in study patients, classified according to outcome.

**Variables**	**Overall**	**Without major aortic events**	**With major aortic event(s)**	***P*-value[a]**
**Number of anti-impulse drug classes**				
baseline	2 (2–3)	2.5 (2–3)	2 (2–3)	0.7
6 months	3 (2–3)	3 (2–3)	2 (2–3)	0.3
12 months	3 (2–3)	3 (2–3)	2 (2–3)	0.4
P-value[b]	0.17	0.17	0.82	
**Number of lipid-lowering drug classes**				
baseline	1 (0–1)	1 (0–1)	1 (0–1)	0.4
6 months	2 (1–2)	2 (1–2)	1 (1–2)	0.7
12 months	2 (1–2)	2 (1–2)	2 (1–2)	0.99
P-value[b]	<0.001*†	<0.001*†	0.008	

Legend. Anti-impulse drugs included beta-blockers, calcium antagonists, angiotensin-converting enzyme inhibitors and angiotensin receptor blockers. Values are reported as median (Q1-Q3). [a]*P*-value for comparison between patients with and without major aortic events. [b]*P*-value of Friedman's test for comparison between time points, in the corresponding patient group. **P* <0.05 for paired comparison between baseline and 6 months; †*P*<0.05 for paired comparison between baseline and 12 months (Wilcoxon test with Holm-Bonferroni correction).

Supplementary Figure 1. Forest plot summarizing the hazard ratio for major aortic events associated with the indicated medical response variables, after correction for abdominal aortic aneurysm presence at inclusion.

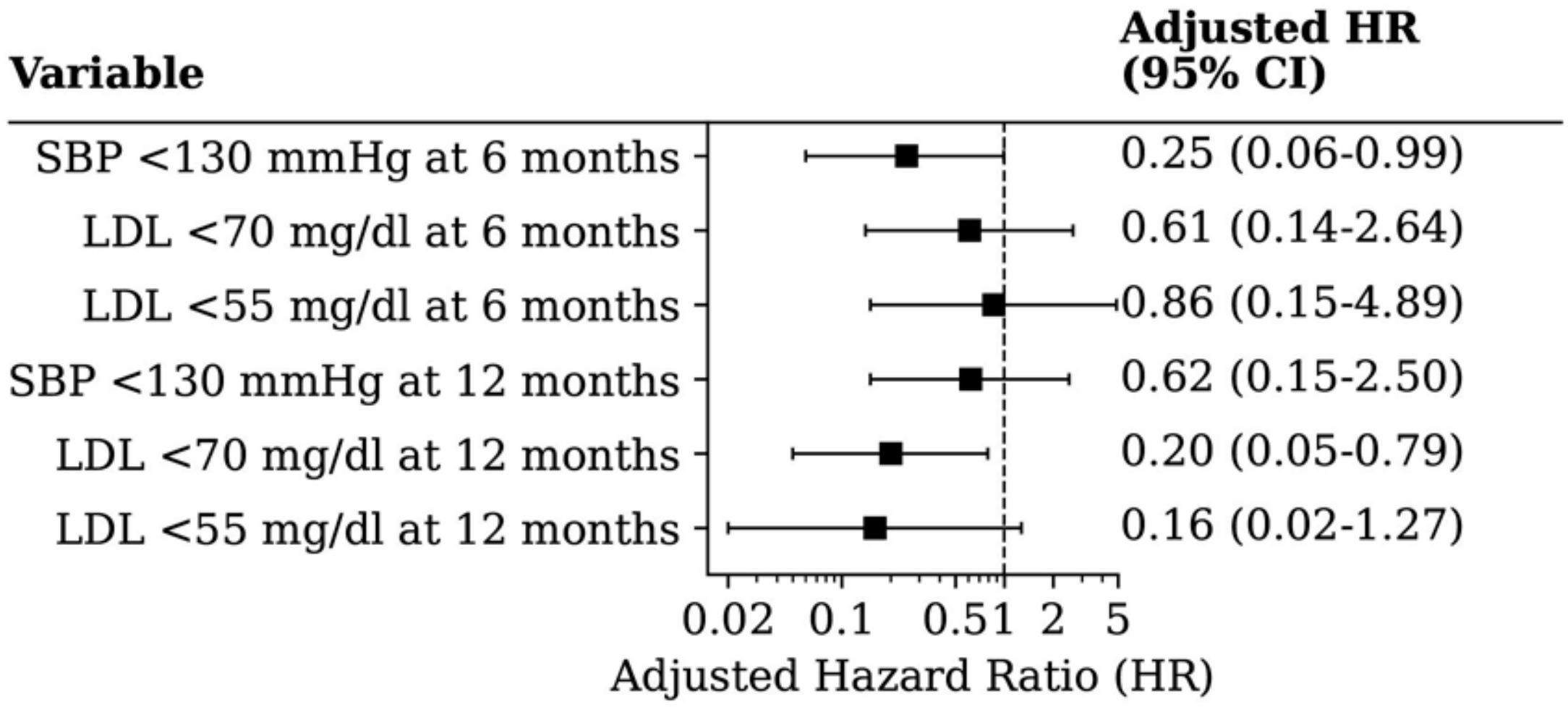

Legend. The adjusted hazard ratio (HR) is presented in log scale. Values in brackets represent the lower and upper limits of the 95% confidence interval.
